# Distinct electrophysiology and dopaminergic modulation of medium spiny neurons across divisions of the tail striatum and the dorsolateral striatum

**DOI:** 10.1113/EP094001

**Published:** 2026-08-21

**Authors:** Bronwyn Riley, Luke E. Hallum, Srdjan M. Vlajkovic, Johanna M. Montgomery, Peter S. Freestone

**Affiliations:** ^1^ Department of Physiology Faculty of Medical and Health Sciences The University of Auckland Auckland New Zealand; ^2^ Faculty of Mechanical and Mechatronics Engineering The University of Auckland Auckland New Zealand

**Keywords:** dopamine, electrophysiology, striatum

## Abstract

The striatum is a brain region within the basal ganglia that plays a crucial role in generating behaviour and mediating perception. The tail striatum (TS) is a poorly understood striatal domain with predominantly sensory functions. Recent evidence supports that abnormal TS dopamine transmission in dopaminergic disease contributes to sensory symptoms by dysregulating TS medium spiny neurons (MSNs). Importantly, the ventral TS is segregated into medial, intermediate and lateral divisions in which TS‐MSNs express D1 and D2 dopamine receptors unevenly, but the functional significance of this is unclear. We aimed to gain insight into the functional significance of this organisation and to investigate how TS‐MSNs are modulated by dopamine. We used whole‐cell patch clamping in rat (P45–55, both sexes) brain slices to conduct the first in‐depth electrophysiological characterisation of TS‐MSNs and compared them across divisions. The adjacent dorsolateral striatum (DLS) was included for comparison. We also investigated how bath‐applied dopamine and selective dopamine agonists SKF38393 and quinpirole modulate MSNs in each region. We found that TS‐MSNs are electrophysiologically distinct from DLS‐MSNs, reflecting the established functional differences between these domains. TS‐MSNs were more depolarised than DLS‐MSNs at rest and had lower rheobase. We also identified functional heterogeneity across the TS divisions. The intermediate division had higher rheobase than the medial or lateral divisions. At rheobase, lateral division TS‐MSNs showed higher firing frequencies than medial or intermediate division TS‐MSNs. Finally, TS‐MSNs showed weaker modulation by dopamine, SKF38393 and quinpirole than DLS‐MSNs, which was also uneven across divisions. These data provide a novel electrophysiological characterisation of TS‐MSNs, showing they are functionally distinct from DLS‐MSNs and heterogeneous across divisions, which could have implications for dopaminergic diseases.

## Abstract (highlights)

- **What is the central question of this study?**
  Are the tail striatum divisions functionally heterogeneous, and how are medium spiny neurons (MSNs) within these divisions modulated by dopamine?
- **What is the main finding and its importance?**
  The first in‐depth electrophysiological characterisation of MSNs in the tail striatum demonstrated that they are functionally heterogeneous across divisions and distinct from MSNs in the dorsolateral striatum. In addition, tail striatal MSNs showed uneven modulation by dopamine and dopamine receptor agonists. These findings advance our functional understanding of the tail striatum and its potential contribution to sensory symptoms in dopaminergic diseases.

## 1 INTRODUCTION

The striatum, as part of the basal ganglia, is a brain region crucial in generating behaviour, including movement, learning, value processing and sensory perception (Lee et al., 2023). Striatal dopamine transmission drives these behaviours by regulating the output of medium spiny neurons (MSNs) through D1 and D2 dopamine receptors (Lanciego et al., 2012). In dopaminergic diseases like Parkinson's disease and schizophrenia, abnormal dopamine transmission dysregulates striatal output and can cause motor and sensory clinical symptoms (Brennan & Arnsten, 2008; Franco et al., 2021; Mosharov et al., 2015)

The motor symptoms of dopaminergic diseases are primarily caused by abnormal dopamine transmission in the dorsolateral striatum (DLS), but the specific mechanisms underlying the sensory symptoms have long been debated (Anderson et al., 2016; Bernheimer et al., 1973; McGregor & Nelson, 2019; Miller et al., 2009). The recent description of a newly identified striatal domain with predominantly sensory functions (the tail striatum; TS) has presented a possible region in which dopamine dysregulation causes sensory symptoms (Gangarossa et al., 2013; Hunnicutt et al., 2016; Miyamoto et al., 2019; Valjent & Gangarossa, 2021). The TS receives dense sensory input (auditory, visual, somatosensory, gustatory, olfactory) from the cortex and thalamus (Hunnicutt et al., 2016) and modulates behaviours associated with sensory cues, including avoidance, engagement, decision‐making and learning (Kintscher et al., 2023).

The TS's unique anatomical features distinguish it from the other striatal domains (Valjent & Gangarossa, 2021). Most strikingly, while D1‐ and D2‐expressing MSNs are intermingled throughout most of the striatum, they are segregated into D1‐rich (medial) and D2‐rich (intermediate) divisions in the TS (Gangarossa et al., 2013). The medial and intermediate divisions, along with a third lateral division containing intermingled D1‐ and D2‐MSNs, occupy the ventral TS in three narrow layers (Miyamoto et al., 2019). The significance of this organisation, and whether the TS divisions are functionally heterogeneous and contribute to distinct behaviours, has not yet been established (Ogata et al., 2022).

Abnormal dopamine transmission in the TS may contribute to sensory symptoms of dopaminergic disease by dysregulating TS MSNs (TS‐MSNs; Schmack et al., 2021; Tsutsui‐Kimura et al., 2025). This is supported by Schmack et al. (2021), who showed that optogenetically stimulating dopamine release in the TS increased the incidence of hallucination‐like behaviours in mice, and this was reversed by the antipsychotic haloperidol. Because of this, advancing our understanding of the TS's modulation by dopamine is important. Here, we used *ex vivo* electrophysiology to characterise the resting and dynamic properties and dopaminergic modulation of MSNs in the medial, intermediate and lateral TS, with comparison to MSNs in the DLS, to gain insight into whether these regions are functionally heterogeneous or homogeneous.

## 2 METHODS

### 2.1 Ethical approval

All experimental procedures were conducted with approval from the animal ethics committee of the University of Auckland (AEC #003472) in accordance with the New Zealand Government Animal Welfare Act. The animal work presented here adheres to the ethical principles regarding animal experiments outlined by *Experimental Physiology*.

### 2.2 Animal housing and husbandry

*Ex vivo* experiments were conducted using brain tissues collected from adult male and female Wistar rats (P50 ± 5). Rats were bred and housed in the Vernon Jansen Unit (University of Auckland, New Zealand). Rats were housed in cages of four or five in a temperature‐ and humidity‐controlled environment with a 12 h dark/12 h light cycle and environmental enrichment. Rats had unrestricted access to food and water up to the time of anaesthesia.

### 2.3 Experimental design

A total of 50 rats were used (31 males and 19 females). No a priori sample size calculations were conducted due to the exploratory nature of this study. However, the sample sizes used are comparable to previous studies characterising the electrophysiology of different neuron types (Baranyi et al., 1993; Schiess et al., 1999). Approximately six brain slices (hemisectioned) were used from each animal, and in each TS brain slice, one recording was typically performed in each division (medial, intermediate, lateral). In experiments involving pharmacological interventions, only one recording was performed in each slice, and paired (within‐subject) comparisons between baseline and drug conditions were made.

### 2.4 Immunostaining and imaging

Rats (*n* = 5) were anaesthetised by intraperitoneal injection of pentobarbitone (100 mg/kg). Once the rats reached deep anaesthesia, as assessed by cessation of movement, significantly slowed breathing, absence of the righting reflex and absence of the toe pinch response, they were transcardially perfused with 100 mL phosphate‐buffered saline (PBS) and then 100 mL paraformaldehyde (PFA; 4%). Rats were then decapitated, and the brain was swiftly removed. A tissue block (ca. 5 mm long) was cut at the optic chiasm and just caudal to the rostral boundary of the pons to obtain a block containing rostral and caudal striatum, which was submerged in PFA. After 24 h, the tissue block was sectioned into 100 µm‐thick brain slices, which were submerged in PBS.

Fixed brain slices were washed in 0.1 M PBS containing 0.5% Triton X‐100 (PBS‐TX) and blocked with PBS‐TX containing 2% bovine serum albumin (A4612, Sigma‐Aldrich, St Louis, MO, USA). Following blocking, slices were incubated overnight at +4°C with a primary antibody mixture (anti‐D1 dopamine receptor antibody, mouse monoclonal; AB78021, Abcam, Waltham, MA, USA) diluted to 1:750 in blocking solution, washed (PBS‐TX, 3 × 5 min), and incubated overnight at room temperature in a secondary antibody mixture (1:500; Alexa fluor 594 goat anti‐mouse IgG; A11032, Thermo Fisher Scientific, Waltham, MA, USA). Slices were then incubated overnight at +4°C with another primary antibody mixture (1:750; anti‐D2 dopamine receptor antibody, rabbit polyclonal; PA5‐115142, Thermo Fisher Scientific), washed (PBS‐TX, 3 × 5 min), and incubated overnight at room temperature in a secondary antibody mixture (1:500; Alexa Fluor 488 donkey anti‐rabbit IgG; A‐21206, Thermo Fisher Scientific). After rinsing, the slices were mounted on glass slides and cover‐slipped with a mounting medium (Citi Fluor AF1; 17970‐25; Electron Microscopy Sciences, Hatfield, PA, USA). The slices were then imaged in brightfield and fluorescence using an inverted microscope (Nikon Eclipse Ti; Nikon, Tokyo, Japan) with appropriate filter sets.

### 2.5 Image analysis

**FIGURE 1:**
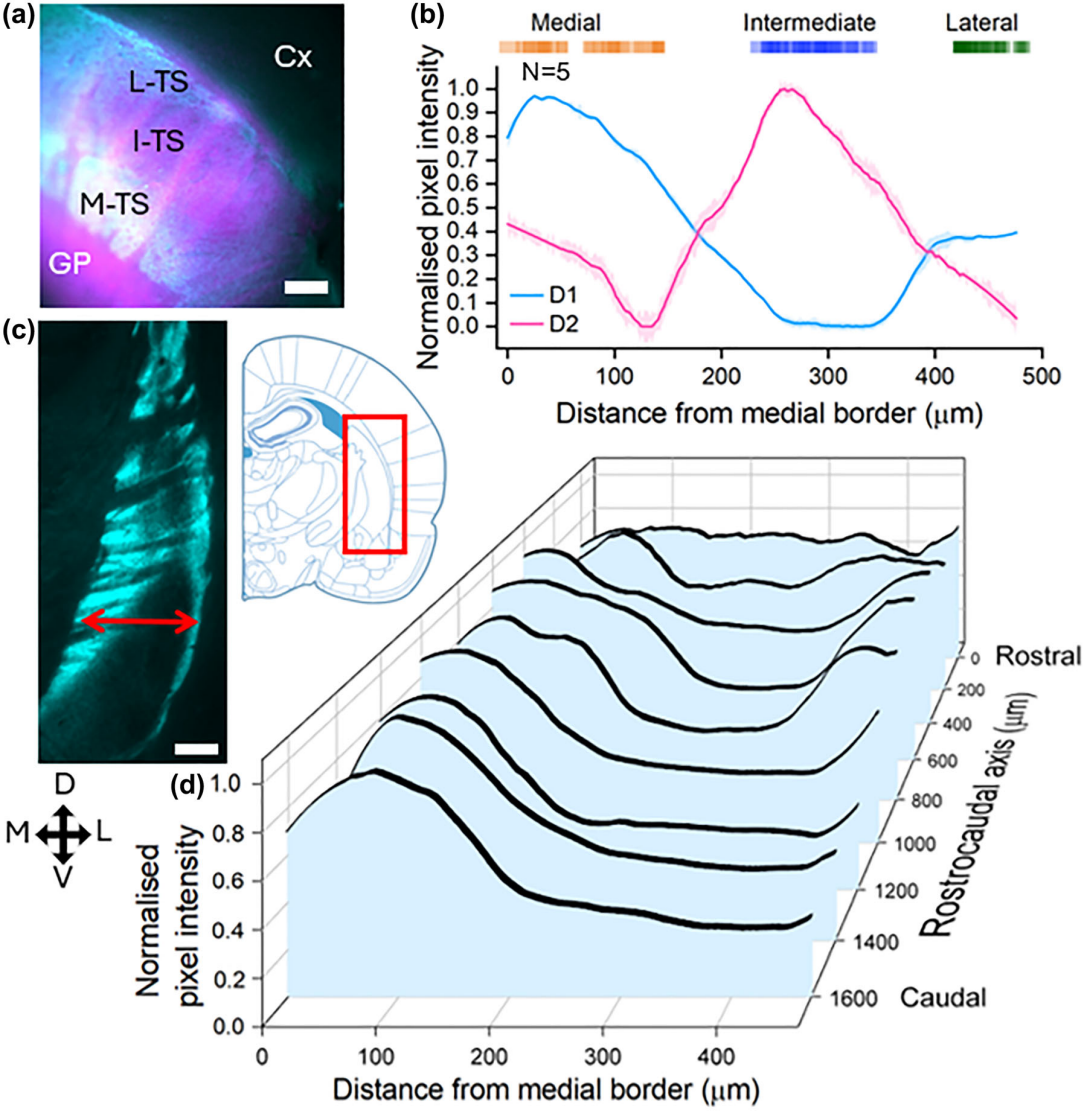
D1 and D2 expression in the tail striatum. (a) Representative image of D1 (cyan) and D2 (magenta) expression in the tail striatum at ×20 magnification; scale bar, 50 µm. Labels indicate the medial, intermediate and lateral divisions of the tail striatum (M‐TS, I‐TS, L‐TS), globus pallidus (GP) and cortex (CX). (b) Normalised line profiles of D1 and D2 fluorescence intensity across the ventral tail striatum averaged over 600 µm along the rostrocaudal axis and 5 rats (15 slices total). Coloured squares show the recording location of patched neurons along the mediolateral axis of the ventral tail striatum. The red arrow in (c) shows the approximate level at which line profiles were taken. (c) Representative image of D1 fluorescence in the tail striatum at ×4 magnification; scale bar, 250 µm. The accompanying schematic (adapted from Paxinos & Watson, 2013) indicates the region images were taken (Bregma –2.16 mm, interaural 6.84 mm). (d) Line profiles of D1 expression along the mediolateral tail striatum in 200 µm steps along the rostrocaudal axis in one rat.

Fluorescence representing D1 or D2 receptor expression was measured in a series of line profiles (medial‐lateral) from each section throughout the TS at the level (dorsoventrally) where electrophysiological recordings would be performed. Line profiles were then averaged over the range of sections (rostro‐caudally) which would be used for electrophysiological recording, covering approximately 1 mm, with five sections per animal (Figure 1b).

The size of the medial, intermediate and lateral divisions in the ventral TS were measured in images of D1 and D2 immunofluorescence. Based on this, we determined that of the medial division covers approximately the most medial 30% of the width of the ventral TS, the intermediate division approximately the central 55%, and the lateral division approximately the most lateral 15%. The position of each recorded neuron on the medial–lateral axis was determined by *post hoc* image analysis of the patch pipette location relative to the borders of the TS (Figure 1b), and MSNs were designated into divisions using the ranges above. After this, the final sample sizes for the three divisions were: medial, 34; intermediate, 46; and lateral, 34.

For each MSN, the long and short axes of the soma were measured in ImageJ Version 1.53e software (Wayne Rasband and National Institutes of Health, Bethesda, MD, USA) from ×40 DIC‐IR images taken of the MSN while patched. Cell images were always taken in the first 1–2 min after patching. The ratio of the short to long axis was reported as an approximation of soma roundness.

### 2.6 *Ex vivo* tissue preparation

Rats (*n* = 45) were deeply anaesthetised by inhalation of isoflurane (ca. 1.0 mL) in an induction chamber prior to performing transcardial perfusion as described previously (Ting et al., 2014) with approximately 30 mL of room temperature carbogenated (5% CO_2_, 95% O_2_) *N*‐methyl‐d‐glutamine (NMDG) artificial cerebrospinal fluid (ACSF; in mM): 100 NMDG, 25 glucose, 20 HEPES, 2.5 KCl, 5 sodium ascorbate, 1.2 NaH_2_PO_4_.2H_2_O, 30 NaHCO_3_, 3 sodium pyruvate, 2 thiourea, 12 *N*‐acetyl cysteine, 0.5 CaCl_2_.2H_2_O, 10 MgSO_4_.7H_2_O (osmolarity 310 mOsm, pH 7.3–7.4). All solutions were continuously carbogenated or saturated with carbogen prior to use. The rat was then decapitated, and the brain was removed and submerged in NMDG‐ACSF. A tissue block containing the rostral and caudal striatum was cut as described in Section 2.2. Coronal brain slices (200 µm) containing the tail or DLS were cut while immersed in ACSF using a Compresstome (VF‐300‐02; Precisionary Instruments, Ashland, MA, USA). Brain slices were then transferred to an incubation chamber warmed to 33°C, and 4 mL NMDG‐ACSF containing 2 M NaCl was pipetted into the chamber in increments over 30 min to slowly reintroduce sodium, as described in Ting et al. (2018). Finally, brain slices were transferred into a holding chamber containing room temperature HEPES‐ACSF (in mM): 100 NaCl, 2.5 KCl, 1.25, NaH_2_PO_4_.2H_2_O, 30 NaHCO_3_, 25 glucose, 20 HEPES, 2 thiourea, 5 sodium ascorbate, 3 sodium pyruvate, 2 CaCl_2_.2H_2_O, 2 MgSO_4_.7H_2_O (ca. 310 mOsm, pH 7.3–7.4) for long‐term incubation. Brain slices were incubated in HEPES‐ACSF for at least 1 h before use.

### 2.7 Electrophysiology

After incubation, individual brain slices were placed in a microscope (Nikon Model Eclipse E600FN; Nikon) recording chamber and perfused (3 mL/min) with room temperature recording ACSF (in mM): 124 NaCl, 2.5 KCl, 1.25 NaH_2_PO_4_.2H_2_O, 26 NaHCO_3_, 2 CaCl_2_.2H_2_O, 2 MgSO_4_.7H_2_O, 12.5 glucose (ca. 310 mOsm, pH 7.3–7.4). Slices were visualised with infrared differential interference contrast (IR‐DIC) at ×4 and ×40 magnification using a CCD camera (ORCA‐ER, Hamamatsu Photonics, Hamamatsu City, Japan) and Micro‐Manager 2.0 software (Micro‐manager; Github, Edelstein et al., 2014). The TS was identified by its characteristic shape and striated appearance based on the rat brain atlas (Paxinos & Watson, 2013) and a previous study (Ogata et al., 2022).

MSNs, which make up 95% of the striatal cell population (Tepper et al., 2010), were identified for whole‐cell patch clamp based on the expected soma size and shape (ovoid, approximately 10–20 µm in length; Jiang & Kim, 2018) under ×40 magnification. Cholinergic neurons, distinguished by their large soma size, were avoided (Kreitzer & Malenka, 2008). Neurons with a maximum evoked firing frequency over 70 Hz were classified as fast‐spiking GABAergic interneurons (Nicola et al., 2000) and were not analysed further. Non‐fast spiking GABAergic interneurons, comprising approximately 1–2% of the striatal cell population in the DLS (Tepper et al., 2010) and TS (Miyamoto et al., 2019), cannot easily be distinguished from MSNs based on electrophysiological and anatomical markers, and may have been included in the analysis. Thus, patched neurons included in the analysis must be defined as putative MSNs (Perk et al., 2021), hereafter referred to as MSNs. As the total number of neurons included in the analysis was 144, approximately two to three interneurons may have been included, given their typical striatal density (Tepper et al., 2010).

Patch pipettes were manufactured using borosilicate glass pipettes (1.2 mm OD, 0.93 mm ID) to have a resistance between 3 and 5 MΩ. Pipettes were filled with an internal solution containing (in mM): 3 ATP, 10 BAPTA, 0.3 Na‐GTP, 10 HEPES, 10 KCl, 112.4 potassium gluconate, 2 MgCl_2_, 10 phosphocreatine–disodium hydrate. A liquid junction potential of 10.3 mV between the recording ACSF and internal solution was measured experimentally, and corrections were applied *post hoc* as described by Barry & Lynch (1991).

### 2.8 Drugs and other treatments

Dopamine hydrocholoride (H8502; Sigma‐Aldrich), SKF‐38393 hydrochloride (S101; Sigma‐Aldrich), quinpirole hydrochloride (Q102; Sigma‐Aldrich), 6‐cyano‐7‐nitroquinoxaline‐2,3‐dione disodium salt hydrate (CNQX; C239; Sigma‐Aldrich), and tetrodotoxin (TTX) with citrate (554412; Sigma‐Aldrich) were formulated as aliquots in milli‐Q water and then diluted to the desired concentration with recording ACSF and bath applied at 3 mL per minute. Dopamine was applied at two concentrations, 30 µM and 100 µM. Quinpirole and SKF38393 were both used at concentrations of 10 µM (Charara & Grace, 2003; Xing et al., 2022). CNQX was used at 20 µM, as per Zhang et al. (2014), and TTX at 1 µM (Chen et al., 2025). All drugs were washed in for 5 min before the data were recorded.

### 2.9 Electrophysiological data analysis

Electrophysiological data were analysed using Clampex version 11 software (Molecular Devices, San Jose, CA, USA) and OriginPro (OriginLab Corp., Northampton, MA, USA). Resting membrane properties (capacitance, membrane resistance, holding current, access resistance, membrane time constant, access resistance) were obtained from the Clampex voltage clamp membrane test (5 mV, 20 ms). Access resistance and holding current were monitored throughout the experiments. Resting membrane potential (RMP) was measured at *I* = 0 (Al‐Muhtasib et al., 2018). Rectifying potassium currents were assessed by applying hyperpolarising voltage steps (–10 mV) starting at –80 mV and were measured from the ratio (inward rectification ratio) of the difference in the holding current between –80 mV and –90 mV and between –120 mV and –130 mV, similar to previous studies (Al‐Muhtasib et al., 2018). Excitatory post‐synaptic currents (EPSCs) were detected using a ClampFit template search and confirmed visually (Forcelli et al., 2012). EPSC frequency and peak amplitude were obtained from the Clampfit results.

Action potential firing frequency was calculated for each depolarising current step (0 to +1000 pA, 1 s) using the total number of action potentials fired during the depolarising pulse and the start and end times of the first and last action potential, respectively. Rheobase was defined as the minimum current injection required to elicit an action potential. Action potential firing was considered to have reached failure when neurons ceased to fire for >50% of the depolarising current injection duration or when successive action potentials decreased in amplitude to <50% of the first action potential fired. The delay to the first action potential was measured at rheobase as the time between the onset of the current step and the onset of the first action potential. The properties of individual action potentials were taken from the first action potential fired at rheobase for each MSN, as done in previous studies (Ting et al., 2024; Willett et al., 2020). Action potentials were analysed using ClampEx threshold event search, and properties (peak amplitude, time to peak, half‐width, and after hyperpolarisation (AHP) amplitude) were obtained from the inbuilt event analysis table.

To compare holding current and EPSC properties between baseline and drug conditions, the last minute of the baseline period was compared to the final minute of the 5 min wash‐in. For comparison of individual action potential properties at rheobase in MSNs where rheobase changed between baseline and dopamine conditions, the action potential properties in dopamine were taken at the new rheobase.

Only recordings with a holding current smaller than –200 pA, access resistance under 20 MΩ, RMP more hyperpolarised than –60 mV and those that fired action potentials when depolarising current was injected were included for analysis. Data from 11 rats were excluded due to poor neuron health which caused neurons to have depolarised RMPs (≤60 mV) and not fire action potentials upon depolarisation, resulting in a final dataset of 34 animals.

### 2.10 Statistical analysis

Regional effects on properties were analysed using the Kruskal–Wallis test in GraphPad Prism 10 software (GraphPad Software, Boston, MA, USA). A non‐parametric test was used due to differences in sample sizes across regions and heterogeneity in variances. Results of the Kruskal–Wallis test were reported using the *P*‐value and Kruskal–Wallis (KW) statistic. Where the regional effect was statistically significant, multiple comparisons were made to compare individual regions. For all multiple comparisons, the two‐stage step‐up method of Benjamini et al. (2006) was used to control the false discovery rate. To investigate the effects of both region and drug application, data were analysed using two‐way repeated‐measures ANOVA, with drug application treated as a within‐subject factor. Where appropriate, multiple comparisons were performed as described previously.

Effect size of region was quantified using Cohen's *d* (Cohen, 1977), which is commonly used in electrophysiological studies (Kashima et al., 2024; Scheuren et al., 2026). Cohen's *d* was calculated from the pooled standard deviation using the following equation from Lakens (2013).

$${\mathrm{Cohen}}^{\prime }s\;d=\frac{X_{1}-X_{2}}{\sqrt{\frac{\left(n_{1}-1\right){\mathrm{SD}}_{1}^{2}+n_{2}-1{\mathrm{SD}}_{2}^{2}}{n_{1}+n_{2}-2}}}$$

In this equation, *X*, *n* and SD denote the mean, sample size and standard deviation, respectively (Lakens, 2013). Cohen's *d* values were interpreted using ranges provided by a previous electrophysiological study (Scheuren et al., 2026): negligible (<0.20), small (0.20–0.50), medium (0.50–0.80) and large (>0.80).

### 2.11 Classification analysis

We used Fisher's linear discriminant analysis to develop a classifier which could determine the TS division a given MSN belonged to (Duda et al., 2000). Our data set comprised the above‐described features (resting membrane properties, EPSC frequency and amplitude, evoked action potential firing properties) derived from the electrophysiological responses of 90 MSNs encountered in the medial (*n* = 28), intermediate (*n* = 38), and lateral (*n* = 24) divisions of the TS (*N* = 20 rats). We developed three binary classifiers: medial versus other, intermediate versus other, and lateral versus other. Features were standardised by subtracting the mean and dividing by the standard deviation across observations. Features were selected using a brute‐force approach, similar to that of Huberty & Olejnik (2006), examining all possible feature subsets of size 3 or 4. We performed this classification with MATLAB (version 23.2.0; MathWorks Inc., Natick, MA, USA).

Leave‐one‐out cross‐validation was used to assess classifiers. To do so, on fold *n*, we used features from all but the *n*‐th recorded MSN to train our classifier and then tested the trained classifier using the features from the *n*‐th recorded MSN, thereby populating 2‐by‐2 confusion matrices (CM). We used CMs to derive accuracy (ACC) and the Matthews correlation coefficient (MCC; Chicco & Jurman, 2020; Matthews, 1975), which is superior to other classifier performance measures when sample sizes are uneven across groups (Chicco & Jurman, 2020). Metrics were calculated in the standard fashion:

$$\mathrm{MCC}=\left(\mathrm{TP}\times \mathrm{TN}-\mathrm{FP}\times \mathrm{FN}\right)\surd \left(\mathrm{TP}+\mathrm{FP}\right)\left(\mathrm{TP}+\mathrm{FN}\right)\left(\mathrm{TN}+\mathrm{FP}\right)\left(\mathrm{TN}+\mathrm{FN}\right)$$

$$\mathrm{ACC}=100\times \left(\mathrm{TN}+\mathrm{TP}\right)/\left(\mathrm{TN}+\mathrm{TP}+\mathrm{FP}+\mathrm{FN}\right)$$

In the equations above, TN, TP, FP and FN denote true negatives, true positives, false positives and false negatives, respectively.

We used a shuffle test to empirically estimate the chance performance of classifiers and create 95% confidence intervals for MCC and ACC based on chance. To do so, we shuffled the class labels in the training set; we then re‐trained and re‐tested the classifier, again using leave‐one‐out cross‐validation. We reasoned that a classifier trained after shuffling labels would be incapable of making meaningful use of features but could nonetheless learn the statistical properties of the dataset and bias its behaviour accordingly (Ojala & Garriga, 2010)

## 3 RESULTS

### 3.1 Immunofluorescence analysis of D1 and D2 expression patterns in the TS

We imaged the TS in five animals using D1 and D2 immunofluorescence to reveal the shape of the TS divisions, which express D1 and D2 unevenly (Figure 1a), at the approximate level of electrophysiological recording (dorsoventrally and rostrocaudally). As expected, we found D1 fluorescence to be brightest in the medial TS and weakest in the intermediate TS, while D2 fluorescence showed the opposite pattern (Figure 1b). This is consistent with Ogata et al. (2022), which showed that D1‐ and D2‐MSNs comprise 80–90% of the medial and intermediate divisions, respectively. We found that the intermediate division occupied most of the ventral TS and had a tear‐drop shape (Figure 1a). The medial division occupied approximately the medial third of the ventral TS, while the lateral division was restricted to a narrow strip (approximately 15%).

We used D1 fluorescence (Figure 1c) to investigate how the shape of the TS divisions changes along the rostrocaudal axis (Figure 1d). In all five animals, the intermediate division emerged as a small area in the middle of the rostral–ventral tail. Moving caudally, it gradually expanded to encompass most of the ventral–lateral tail and extend dorsally along the length of the TS's midline. This expression pattern is generally consistent with prior reports of the intermediate division's shape along the rostrocaudal axis imaged using tyrosine hydroxylase immunohistochemistry in mice (Miyamoto et al., 2019; Ogata et al., 2022).

### 3.2 Electrophysiological analysis of TS‐MSNs

#### 3.2.1 Resting membrane and size properties of TS‐MSNs

The electrophysiological properties of DLS‐MSNs have been characterised extensively (Al‐Muhtasib et al., 2018; Gertler et al., 2008). However, the properties of TS‐MSNs remain poorly understood. Our results presented here expand on the only previous study that reported the electrophysiology of TS‐MSNs (Ogata et al., 2022).

We found that TS‐ and DLS‐MSNs had distinct membrane and size properties. RMP, capacitance and the related property, membrane time constant (tau), were significantly lower in TS‐MSNs than in DLS‐MSNs (Table 1). TS‐MSN somas were also larger and more rounded than DLS‐MSNs, with similar long axis lengths but significantly larger short axis lengths. DLS‐MSNs here were consistent with previous studies using similar preparations (Al‐Muhtasib et al., 2018; Gertler et al., 2008; Jiang & Kim, 2018; Planert et al., 2013; Tepper et al., 1998; Venance & Glowinski, 2003).

**TABLE 1:** Resting membrane and size properties of MSNs in the dorsolateral striatum (DLS) and tail striatum (TS) divisions.

	Medial TS (*n* = 34)	Intermediate TS (*n* = 46)	Lateral TS (*n* = 34)	DLS (*n* = 30)	Kruskal–Wallis test	Multiple comparisons *P*‐value relative to the DLS	
Long axis length (µm)	10.2 ± 2.0	11.2 ± 2.3	10.9 ± 3.1	10.5 ± 1.8	*P =* 0.254 KW = 4.07 NS	N/A	
Short axis length (µm)	7.6 ± 1.8	8.4 ± 2.0	7.9 ± 1.8	5.9 ± 1.2	*P <* 0.0001 KW = 25.2 ^**^	M	0.00134
						I	<0.0001
						L	0.000256
Short axis: long axis ratio	0.75 ± 0.14	0.74 ± 0.15	0.72 ± 0.16	0.61 ± 0.15	*P =* 0.0495 KW = 7.50 ^*^	M	0.00161
						I	0.00972
						L	0.0469
RMP (mV)	−72.3 ± 6.5	−74.0 ± 7.2	−73.3 ± 7.4	−78.8 ± 5.7	*P =* 0.00781 KW = 11.9 ^**^	M	0.00159
						I	0.0147
						L	0.00336
Capacitance (pF)	40.7 ± 27.0	41.08 ± 31.8	37.3 ± 25.4	65.8 ± 25.6	*P =* 0.0194 KW = 9.90 ^*^	M	0.0192
						I	0.0101
						L	0.0120
Membrane resistance (MΩ)	248 ± 174	175 ± 107	195 ± 100	211 ± 115	*P =* 0.286 KW = 3.80 NS	N/A	
Tau (ms)	833 ± 701	703 ± 641	814 ± 714	1255 ± 587	*P =* 0.00257 KW = 14.2 ^**^	M	0.0122
						I	0.000710
						L	0.00644
Inward rectification ratio	1.61 ± 0.40 *n* = 20	1.90 ± 0.66 *n* = 28	1.81 ± 0.65 *n* = 23	1.89 ± 0.83 *n* = 20	*P =* 0.544 KW = 2.10 NS	N/A	
Δ current for −10 mV step (pA)	−73.8 ± 47.4	−79.9 ± 47.3	−57.9 ± 20.6	−83.2 ± 52.7	*P =* 0.804 KW = 1.02 NS	N/A	

*Note*: *n* values representing the number of cells are shown in the first row or in the sixth row for the sixth and seventh rows. TS properties were recorded from *N* = 34 rats and DLS properties were recorded from *N* = 25 rats. TS inward rectification properties were recorded in *N* = 20 rats and DLS inward rectification properties were recorded in *N* = 15 rats. Data presented as means ± SD. Regional comparisons were made with Kruskal–Wallis tests, and the *P*‐value and Kruskal–Wallis (KW) statistic reported. (NS *P >* 0.05, *P < 0.05, **P < 0.01, ***P < 0.0001). When significant, multiple comparisons *P*‐values relative to the DLS were also reported. No significant differences were identified between TS divisions (*P >* 0.05).

**FIGURE 2:**
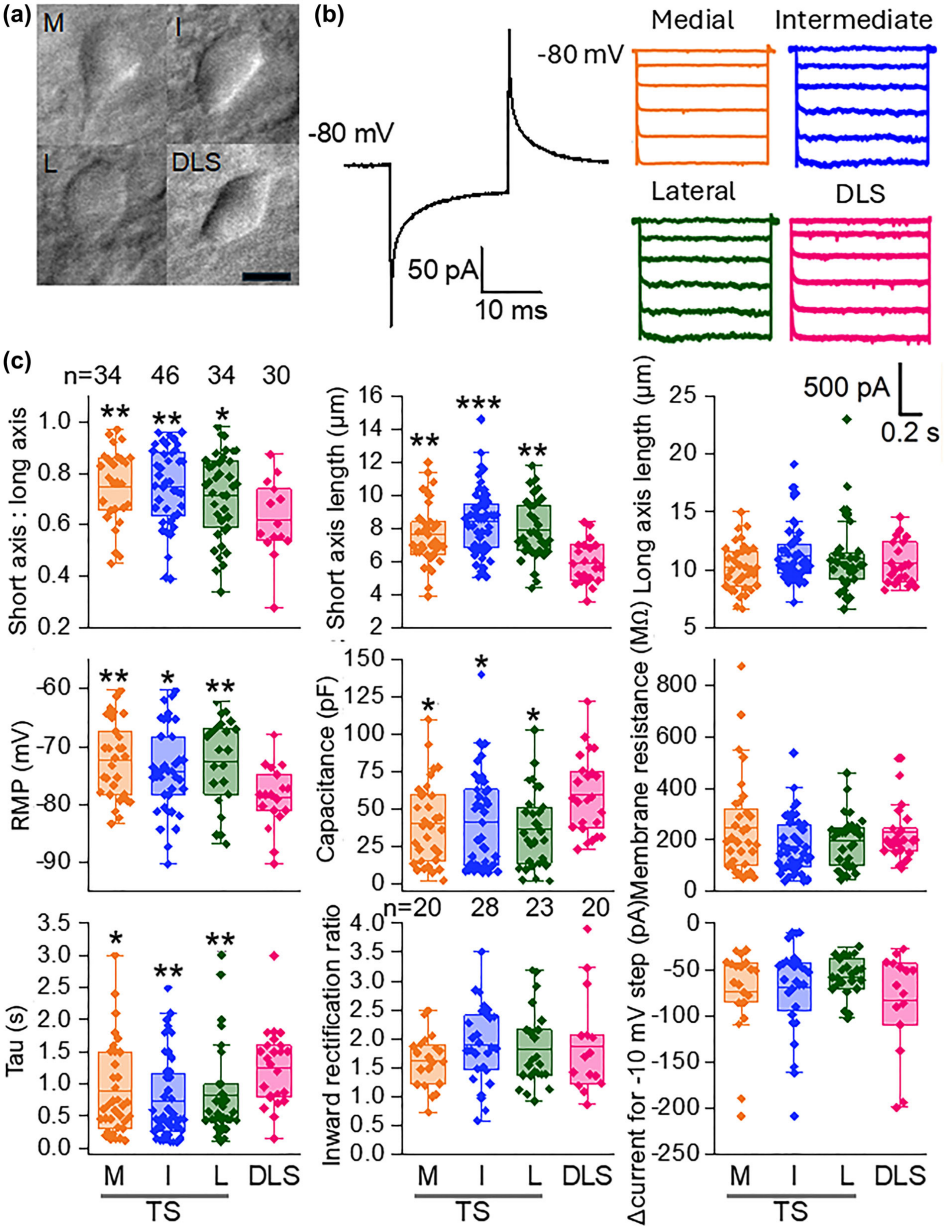
Resting membrane and size properties of MSNs in the dorsolateral striatum (DLS) and tail striatum (TS) divisions (medial, M; intermediate, I; lateral, L). (a) Representative images of MSN somas in each region at ×40 magnification. Scale bar: 10 µm. (b) Left: representative trace (average of 10 sweeps) of one TS‐MSN response to the membrane test (−5 mV, 20 ms step) from which resting membrane properties were calculated. Right: representative traces of current responses to hyperpolarising steps from rest (−80 mV to −130 mV) from one MSN in each region. (c) Resting membrane and size properties in each region. *n* values represent the number of cells: M, 34; I, 46; L, 34 (*N* = 34 rats); DLS, 30 (*N* = 25 rats). Rectifying current properties: M, 20; I, 28; L, 23 (*N* = 20 rats); DLS, 20 (*N* = 15 rats). Regional comparisons were made with Kruskal–Wallis tests and significant multiple comparisons shown. *Significant difference in comparison to the DLS (*P < 0.05, **P < 0.01, ***P < 0.0001). No significant differences were identified between TS divisions (*P >* 0.05).

While electrophysiological differences were identified between TS‐ and DLS‐MSNs, both regions generally retained key characteristic properties of MSNs (Table 1; Figure 2), including ovoid somas between 10 and 20 µm in length, low membrane resistance and strong rectifying currents (Al‐Muhtasib et al., 2018). However, TS‐MSNs had more depolarised RMPs (–73 ± 1 mV, *n* = 114 cells from *N* = 34 rats) than is typical for MSNs (approximately –80 mV; Planert et al., 2013), while DLS‐MSNs had RMPs in the typical range (–78.8 ± 1.3 mV, *n* = 30 cells from *N* = 25 rats). Conversely, one previous study (Ogata et al., 2022), which investigated membrane properties in a small sample of TS‐MSNs, reported that MSNs in the medial and intermediate TS (lateral division not investigated) had more typical RMPs of approximately –78 mV, but in mice.

We found no significant variation in inward rectification ratio (Table 1), which reflects the strength of rectifying potassium currents, a powerful determinant of RMP (Nisenbaum & Wilson, 1995). We also identified no significant regional differences in the current required to step from –80 mV to –90 mV, reflecting the strength of rectifying currents close to RMP.

We found no statistically significant differences (Appendix, Table A1) in TS‐MSN soma size, nor resting membrane properties across divisions (Table 1; Figure 2). Ogata et al. (2022) also found no differences in membrane properties between the medial and intermediate divisions but did not investigate the lateral division.

Sex had no statistically significant effect on electrophysiological properties as determined by non‐parametric Mann‐Whitney *U*‐tests (all *P >* 0.1). Only one property (capacitance) showed a significant correlation with age, as determined by Pearson's correlation test (*r* = 0.32, *P* = 0.005). Neurons from rats of different ages were evenly distributed across regions (Kruskal–Wallis test; *P >* 0.05; KW = 0.14), so this is unlikely to bias regional comparisons.

#### 3.2.2 Spontaneous EPSCs

**FIGURE 3:**
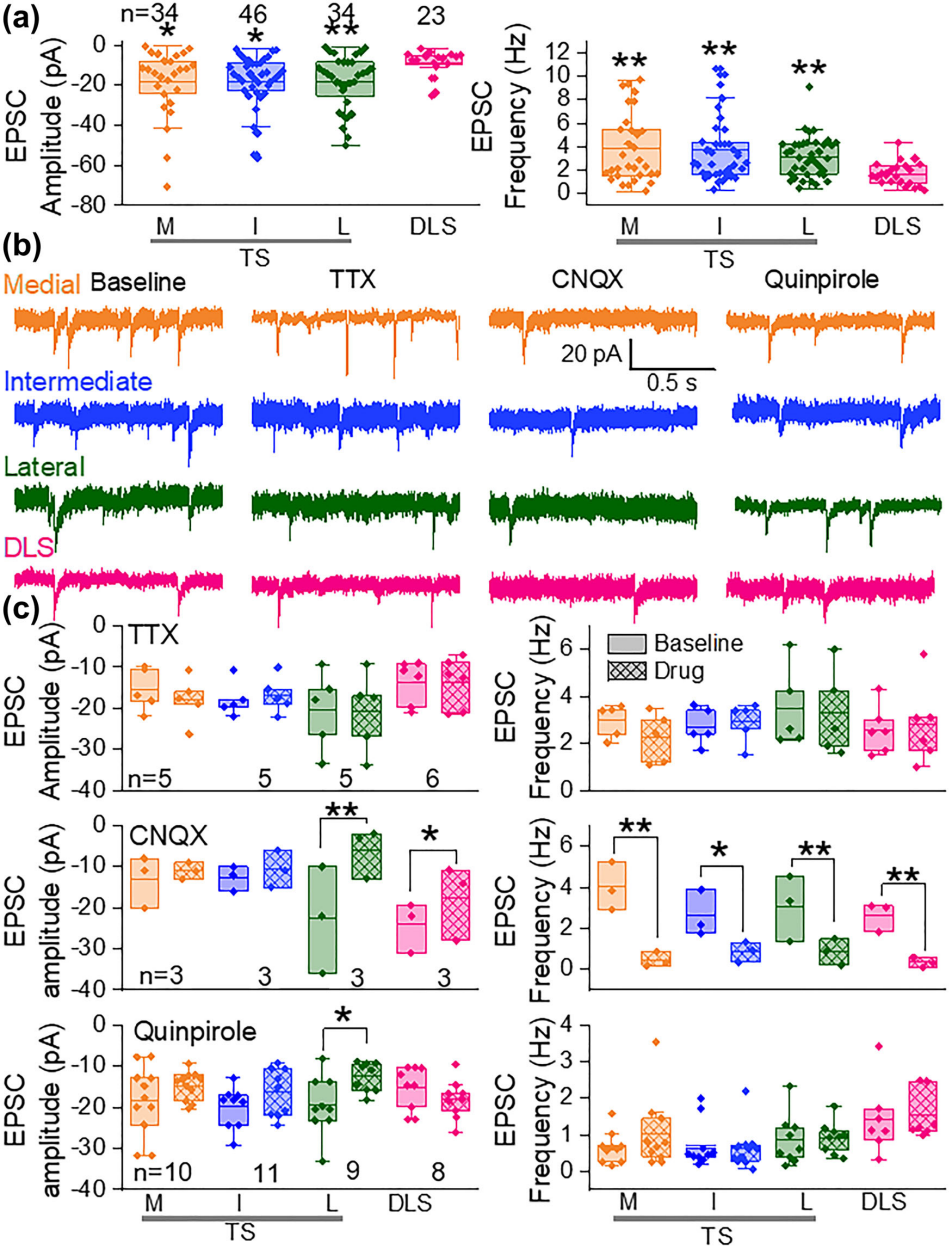
Spontaneous excitatory postsynaptic currents (EPSC) in the dorsolateral striatum (DLS) and tail striatum (TS) divisions (medial, M; intermediate, I; lateral, L). (a) EPSC amplitude and firing frequency in baseline conditions in all regions. *n* values representing the number of cells: M, 34; I, 46; L, 34; DLS, 23. TS data were recorded in *N* = 34 rats and DLS data were recorded in *N* = 20 rats. Regional comparisons were made with Kruskal–Wallis tests and significant multiple comparisons shown. *Significant difference in comparison to the DLS. (b) Representative traces of EPSCs recorded in voltage clamp (−80 mV) in baseline conditions and in the presence of tetrodotoxin (TTX; 1 µM), cyanquixaline (CNQX; 20 µM) or quinpirole (10 µM) from one MSN in each region. (c) EPSC amplitude and firing frequency in baseline conditions and following drug application in all regions. *n* values representing the number of cells: TTX (*N* = 5 rats): M, 5; I, 5; L, 5; DLS, 6. CNQX (*n* = 3 rats): M, 3; I, 3; L, 3; DLS, 3. Quinpirole (*N* = 8 rats): M, 10; I, 11; L, 9; DLS, 8. Statistical significance of drug effect was tested using repeated‐measures two‐way ANOVA and significant within‐region effects as per multiple comparisons are shown (*P < 0.05, **P < 0.01, ***P < 0.0001).

Glutamatergic terminals from the cortex and thalamus synapse with MSNs in the striatum and, when activated, create excitatory post‐synaptic currents (EPSC; Willett et al., 2019). Here, spontaneous EPSCs occurred consistently in all MSNs during voltage‐clamp recordings at −80 mV (Figure 3). Interestingly, spontaneous EPSCs occurred at a significantly higher frequency in TS‐MSNs than in DLS‐MSNs, by approximately 2‐fold (Table 2). TS‐MSNs also fired significantly larger amplitude EPSCs than DLS‐MSNs. No statistically significant differences in spontaneous EPSC frequency or amplitude were identified between the TS divisions (Appendix, Table A1).

**TABLE 2:** Spontaneous excitatory postsynaptic current (EPSC) properties in the dorsolateral striatum (DLS) and tail striatum (TS) divisions.

	Medial TS (*n* = 34)	Intermediate TS (*n* = 46)	Lateral TS (*n* = 34)	DLS (*n* = 23)	Kruskal–Wallis test	Multiple comparisons *P*‐values relative to the DLS	
EPSC amplitude (pA)	−18.6 ± 16.6	−18.1 ± 13.0	−18.2 ± 13.2	−8.9 ± 6.4	*P =* 0.0306 KW = 8.90 ^*^	M	0.0341
						I	0.0186
						L	0.00926
EPSC frequency (Hz)	3.81 ± 2.88	3.77 ± 2.77	3.11 ± 1.76	1.69 ± 0.95	*P =* 0.00375 KW = 13.4 ^**^	M	0.00400
						I	0.00193
						L	0.00426

*Note*: *n* values representing the number of cells and are shown in the first row. TS properties were recorded in *N* = 34 rats and DLS properties were recorded in *N* = 25 rats. Data presented as means ± SD. Regional comparisons were made with Kruskal–Wallis tests and the *P*‐value and Kruskal–Wallis (KW) statistic reported. (NS *P >* 0.05, *P < 0.05, **P < 0.01, ***P < 0.0001). When significant, multiple comparisons *P*‐values relative to the DLS were also reported. No significant differences were identified between TS divisions (*P >* 0.05).

TTX (1 µM; Table 3, Figure 3) caused no change in spontaneous EPSC frequency or amplitude in the TS or DLS (spontaneous EPSCs were equivalent to miniature EPSCs; Hohnke & Sur, 1999). This is consistent with a previous study of dorsal striatum MSNs, which also used rat striatal slices (Tang & Lovinger, 2000). Our findings indicate that action potential‐mediated events arising from long‐distance corticostriatal connections do not contribute meaningfully to total spontaneous EPSCs in the TS or DLS because these connections are severed during brain slice preparation (Sun et al., 2013).

**TABLE 3:** Spontaneous excitatory postsynaptic current (EPSC) properties in the dorsolateral striatum (DLS) and tail striatum (TS) divisions in the presence of 1 µM tetrodotoxin (TTX), 20 µM cyanquixaline (CNQX) and 10 µM quinpirole.

	Medial TS	Intermediate TS	Lateral TS	DLS	Two‐way ANOVA
TTX EPSC amplitude (pA) (*N* = 5 rats)	−17.6 ± 4.3 *n* = 5	−16.8 ± 4.3 *n* = 5	−20.9 ± 8.6 *n* = 5	−11.14 ± 7.6 *n* = 6	*P =* 0.828 *F*(1, 31) = 0.047 NS
TTX EPSC frequency (Hz)	3.0 ± 0.8	3.9 ± 0.8	3.1 ± 1.5	1.6 ± 0.6	*P =* 0.690 *F*(1, 31) = 0.166 NS
CNQX EPSC amplitude (pA) (*N* = 3 rats)	−11.0 ± 1.1 *n* = 3	−10.7 ± 2.6 *n* = 3	−17.7 ± 5.2 *n* = 3	−6.0 ± 2.3 *n* = 3	*P =* 0.0311 *F*(1, 16) = 5.53 ^*^
Within‐region *P*‐value	0.6818	0.6912	0.0056	0.0202	
CNQX EPSC frequency (Hz)	0.5 ± 0.2	0.9 ± 0.4	0.9 ± 0.2	0.3 ± 0.1	*P* ≤ 0.0001 *F*(1, 16) = 44.5 ^**^
Within‐region *P*‐value	0.000104	0.0101	0.00291	0.00223	
Quinpirole EPSC amplitude (pA) (*N* = 8 rats)	−15.2 ± 2.9 *n* = 10	−20.0 ± 9.3 *n* = 11	−15.1 ± 3.1 *n* = 9	−18.8 ± 2.2 *n* = 8	*P =* 0.0292 *F*(1, 32) = 6.01 ^*^
Within‐region *P*‐value	0.2262	0.2165	0.0129	0.5987	
Quinpirole EPSC frequency (Hz)	3.1 ± 2.8	1.7 ± 0.7	2.7 ± 1.2	4.5 ± 1.8	*P =* 0.549 *F*(1, 32) = 0.367 NS

*Note*: *n* values representing the number of cells are shown in the first row for each condition and *N* values representing the number of rats in which data were recorded are shown in the first column for each condition. Data presented as means ± SD. The statistical significance of the drug effect was tested using repeated‐measures two‐way ANOVA, and the *P‐* and *F‐*values reported. (NS *P >* 0.05, *P < 0.05, **P < 0.01, ***P < 0.0001). When significant, within‐region *P*‐values as per multiple comparisons were also reported.

Application of the AMPA/kainate antagonist CNQX (20 µM) significantly reduced EPSC frequency by > 75% in the DLS and TS divisions (Table 3, Figure 3). This shows that in all regions, the majority of the events recorded at baseline are mediated by AMPA/kainate receptors (Jeun et al., 2009). Remaining events are likely GABAergic IPSCs (Arama et al., 2015). CNQX also significantly reduced EPSC amplitude, but only in the DLS and lateral TS, as per multiple comparisons. These findings in the DLS are consistent with previous work (Jeun et al., 2009).

EPSCs can be modulated by dopamine through D2 receptors expressed on thalamocortical terminals. This is quite rare in the dorsal striatum but more common in other parts of the striatum, such as the nucleus accumbens (Brundege & Williams, 2002). We found that D2‐agonism with quinpirole (10 µM) significantly reduced EPSC amplitude (Table 3). However, multiple comparisons showed that this was only statistically significant in the lateral division (Table 3), where EPSC amplitude was reduced by 30 ± 7% (*n* = 9 cells from *N* = 8 rats). Quinpirole did not have a significant effect on EPSC frequency.

Dopamine (30 µM and 100 µM) and D1‐agonism with SKF38393 did not significantly affect EPSC frequency or amplitude (Appendix, Table A2).

Using these voltage clamp recordings, we additionally confirmed that dopamine (30 µM and 100 µM), SKF38393 and quinpirole had no significant effect on holding current, reflecting membrane potential (dopamine 30 µM, *P* = 0.62, *F*(1, 45) = 0.24; dopamine 100 µM, *P* = 0.65, *F*(1, 61) = 0.20; SKF38393, *P* = 0.16, *F*(1, 31) = 0.20; quinpirole *P* = 0.37; *F*(1, 32) = 0.24).

#### 3.2.3 Action potential firing and kinetics

**FIGURE 4:**
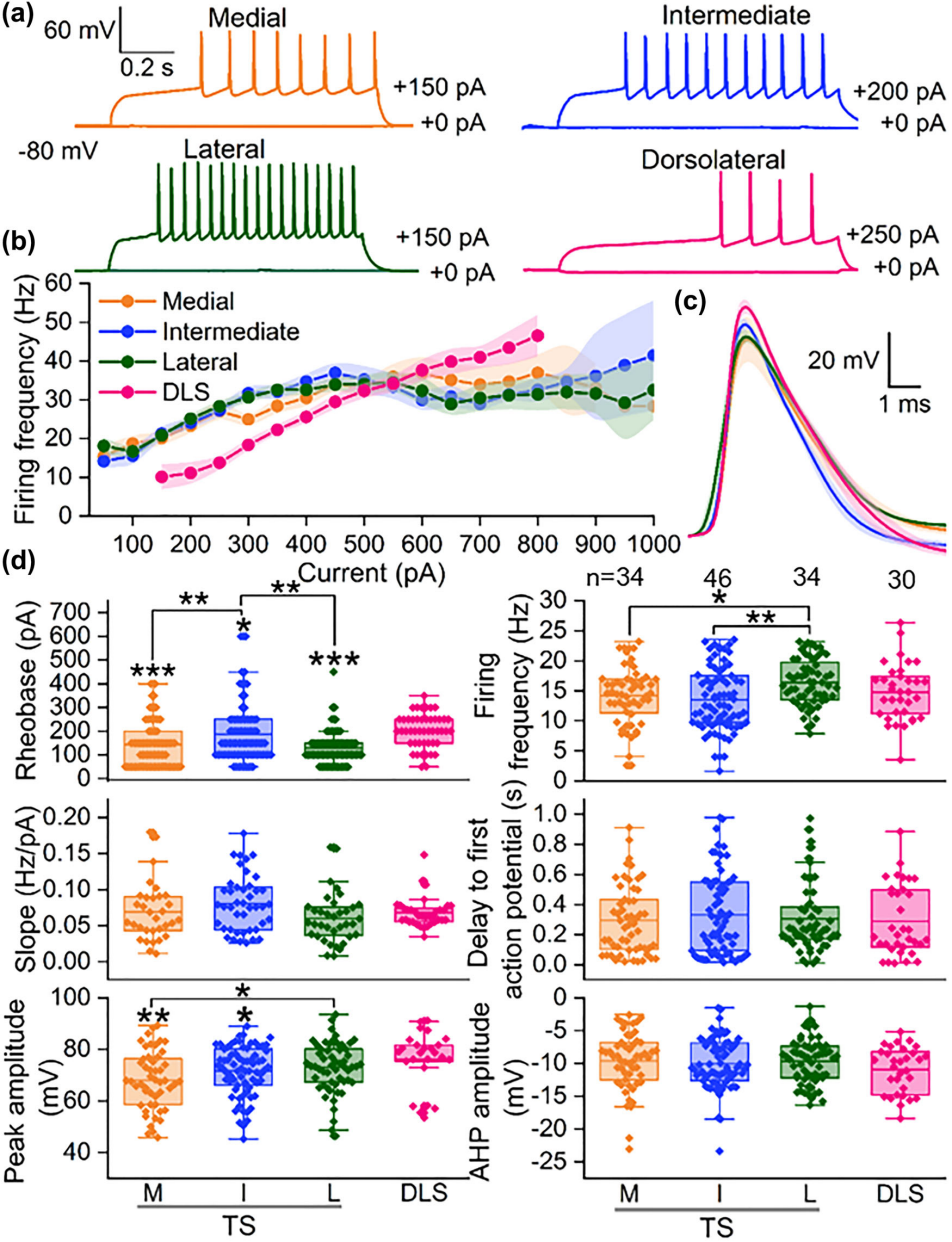
Evoked action potential firing properties in the dorsolateral striatum (DLS) and tail striatum (TS) divisions (medial, M; intermediate, I; lateral, L). (a) Representative traces of action potential firing at rheobase from one MSN in each region. (b) Action potential firing frequency over increasingly depolarising current injections. *n* values representing the number of cells: M, 34; I, 46; L, 34; DLS, 30. TS properties were recorded in *N* = 34 rats and DLS properties were recorded in *N* = 25 rats. (c) First action potential fired at rheobase (average + error) for each region. (d) Evoked action potential firing properties in all regions. Firing frequency and delay to first action potential were measured at rheobase. Action potential shape properties were measured from the first action potential fired at rheobase. Regional comparisons were made with Kruskal–Wallis tests and significant multiple comparisons shown. Asterisk denotes a significant difference in comparison to the DLS; asterisk with a bar denotes a significant difference between TS divisions (*P < 0.05, **P < 0.01, ***P < 0.0001).

We evoked action potential firing in MSNs by applying depolarising current steps from 0 pA to 1000 pA while neurons were current clamped at −80 mV. TS‐MSNs exhibited firing patterns typical of MSNs elsewhere in the striatum, including a prolonged delay to first action potential, regular firing rate and prominent AHP amplitude (Table 4, Figure 4; Belleau & Warren, 2000; Klapstein et al., 2001; Ting et al., 2024)

**TABLE 4:** Evoked action potential firing properties of MSNs in the dorsolateral striatum (DLS) and tail striatum (TS) divisions.

	Media TS (*n* = 34)	Intermediate TS (*n* = 46)	Lateral TS (*n* = 34)	DLS (*n* = 30)	Kruskal–Wallis test	Multiple comparisons *P*‐values relative to the DLS	
Rheobase (pA)	143 ± 94	188 ± 122	128 ± 58	202 ± 70	*P <* 0.0001 KW = 33.1 ^***^	M	<0.0001
						I	0.0232
						L	<0.0001
Firing frequency (Hz)	14.2 ± 4.8	13.5 ± 5.2	16.4 ± 3.8	14.8 ± 4.7	*P =* 0.0101 KW = 11.3 ^*^	M	>0.999
						I	>0.999
						L	0.657
Delay to first action potential (s)	0.29 ± 0.22	0.33 ± 0.26	0.30 ± 0.22	0.29 ± 0.23	*P =* 0.852 KW = 7.89 NS	N/A	
Slope (Hz/pA)	0.07 ± 0.04	0.08 ± 0.05	0.06 ± 0.04	0.07 ± 0.04	*P =* 0.219 KW = 4.42 NS	N/A	
Peak amplitude (mV)	68.0 ± 11.2	72.0 ± 9.6	73.0 ± 9.7	75.7 ± 10.8	*P =* 0.0102 KW = 11.3 ^*^	M	0.00604
						I	0.0498
						L	0.147
Time to peak (ms)	1.60 ± 0.46	1.45 ± 0.43	1.50 ± 0.46	1.45 ± 0.34	*P =* 0.229 KW = 4.32 NS	N/A	
Half‐width (ms)	3.1 ± 1.5	2.9 ± 1.4	3.2 ± 1.6	2.2 ± 0.9	*P =* 0.291 KW = 3.71 NS	N/A	
AHP amplitude (mV)	−9.6 ± 4.3	−10.0 ± 3.8	−9.7 ± 3.4	−10.9 ± 3.4	*P =* 0.402 KW = 2.93 NS	N/A	

*Note*: Firing frequency and delay to first action potential were measured at rheobase. Action potential shape properties were measured from the first action potential fired at rheobase. *n* values representing the number of cells are reported in the first row. TS properties were recorded in *N* = 34 rats and DLS properties were recorded in *N* = 25 rats. Data presented as means ± SD. Regional comparisons were made with Kruskal–Wallis tests and the *P*‐value and Kruskal–Wallis (KW) statistic reported. (NS > 0.5, *P < 0.05, **P < 0.01, ***P < 0.0001). When significant, multiple comparisons *P*‐values relative to the DLS were also reported. Multiple comparisons *P*‐values between TS divisions are shown in Table 5.

We found rheobase to be significantly higher in the DLS than in the TS across all divisions (Table 4). Conversely, Ogata et al. (2022) reported higher rheobase in the DLS than in the intermediate TS, but not the medial TS. We also found TS‐MSNs in the medial and intermediate divisions, but not the lateral division, to have significantly smaller action potential peak amplitude relative to DLS‐MSNs.

Importantly, we identified statistically significant variation in firing properties across the TS divisions (Table 4; Figure 4). TS‐MSNs in the intermediate division had significantly higher rheobase than TS‐MSNs in the lateral and medial divisions. TS‐MSNs in the lateral division fired at higher frequencies at rheobase than TS‐MSNs in the medial or intermediate divisions. Finally, TS‐MSNs in the lateral division had higher action potential peak amplitudes than TS‐MSNs in the medial division. Firing properties with otherwise consistent across divisions (Appendix, Table A1).

To better understand the magnitude of the variation in firing properties across the TS divisions, we calculated the effect size (Cohen's *d*; Table 5) of properties which showed statistically significant differences between TS division pairs (Cohen, 1977). Region showed a small effect on the difference in rheobase between the medial and intermediate divisions, and a medium effect between the intermediate and lateral divisions. Region had a medium effect on the difference in firing frequency between the medial and lateral divisions and between the intermediate and lateral division. Finally, the effect of region on the difference in peak amplitude between the medial and lateral divisions was small. Overall, these findings indicate that the TS divisions exhibit small to moderate differences in action potential firing properties. In contrast, Ogata et al. (2022) found no statistically significant differences in firing properties between the medial and intermediate divisions, but with smaller sample sizes than were used here.

**TABLE 5:** Paired regional comparisons of action potential firing properties between tail striatum (TS) divisions.

	TS division pair	Multiple comparisons *P*‐value	Cohen's *d* effect size	Interpretation
Rheobase	Medial and intermediate	0.00681	0.399	Small
	Intermediate and lateral	0.000700	0.646	Medium
Firing frequency	Medial and lateral	0.0196	0.513	Medium
	Intermediate and lateral	0.00100	0.601	Medium
Peak amplitude	Medial and lateral	0.0258	0.479	Small

*Note*: Only TS division pairs in which a statistically significant difference (*P <* 0.05) was identified in multiple comparisons following significant Kruskal–Wallis tests are included and the *P*‐value reported. The effect size (Cohen's *d*) of region on the variation of these properties between region pairs is also shown. Ranges for interpretation were based on a previous study (Scheuren et al., 2026) and were as follows: negligible (<0.20), small (0.20–0.50), medium (0.50–0.80) and large (>0.80).

#### 3.2.4 Modulation of action potential firing by dopamine

How dopamine modulates action potential firing across the TS divisions is of interest, given their uneven expression of D1 and D2 (Ogata et al., 2022), and evidence implicating dopaminergic modulation of TS‐MSNs in sensory symptoms of disease (Schmack et al., 2021). We compared action potential firing in baseline conditions and in the presence of dopamine (30 µM and 100 µM). Dopamine altered MSN firing properties in all TS divisions and in the DLS, but the pattern of dopaminergic modulation in each region was distinct.

**FIGURE 5:**
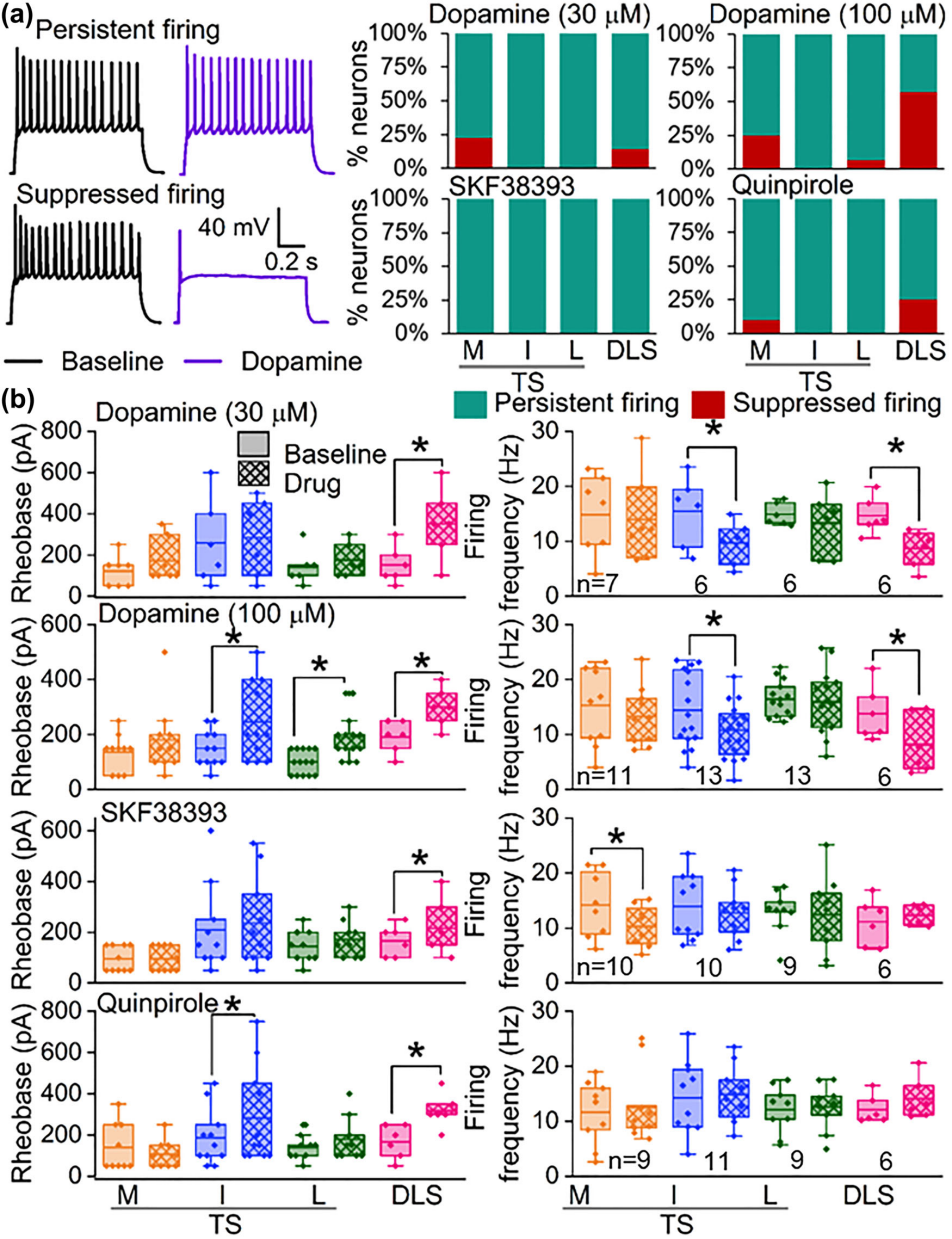
Modulation of evoked action potential firing by dopamine (30 µM and 100 µM), SKF38393 (10 µM) and quinpirole (10 µM) in the dorsolateral striatum (DLS) and tail striatum (TS) divisions (medial, M; intermediate, I; lateral, L). (a) Left: example traces of persistent and suppressed firing responses from individual neurons in the medial tail striatum. Right: distribution of suppressed and persistent action potential firing responses to drug application. *n* values representing the number of cells: Dopamine 30 µM (*N* = 6 rats): M, 9; I, 6; L, 6; DLS, 7. Dopamine 100 µM (*N* = 11 rats): M, 12; I, 13; L, 14; DLS, 14. SKF38393 (*N* = 7 rats): M, 10; I, 10; L, 9; DLS, 7. Quinpirole (*N* = 8 rats): M, 10; I, 11; L, 9; DLS, 8. (b) Paired comparison of action potential firing properties in baseline and drug conditions. Only persistent firing neurons are included. n values representing the number of cells: Dopamine 30 µM: M, 7; I, 6; L, 6; DLS, 6. Dopamine 100 µM: M, 9; I, 13; L, 13; DLS, 6. SKF38393: M, 10; I, 10; L, 9; DLS, 7. Quinpirole; M, 9; I, 11; L, 9; DLS, 6. Within‐region comparisons of drug effects were made using repeated‐measures two‐way ANOVA and significant within‐region effects as per multiple comparisons shown (*P < 0.05, **P < 0.01, ***P < 0.0001).

We found that dopamine (30 µM and 100 µM) powerfully suppressed action potential firing in a subset of MSNs, causing them to fire only a single action potential per current step. This firing suppression was most common in the DLS (Table 6, Figure 5), particularly following application of 100 µM dopamine. Dopamine‐induced firing suppression in the DLS has been reported previously, affecting a similar proportion of MSNs (Akaike et al., 1987; Brown & Arbuthnott, 1983; Calabresi et al., 1987). Dopamine also induced firing suppression in some TS‐MSNs, primarily in the medial division, which, like the DLS, showed suppressed firing at both 30 µM and 100 µM dopamine (Table 6, Figure 5). In comparison, the lateral division showed only minimal firing suppression by 100 µM dopamine, and the intermediate division did not show firing suppression at either concentration.

**TABLE 6:** Proportion of MSNs in the dorsolateral striatum (DLS) and tail striatum (TS) divisions showing firing suppression in response to dopamine (30 µM and 100 µM), SKF38393 (10 µM) and quinpirole (10 µM).

	Medial TS	Intermediate TS	Lateral TS	DLS
30 µM dopamine (*N* = 6 rats)	2/9	0/6	0/6	1/7
100 µM dopamine (*N* = 11 rats)	3/12	0/13	1/14	8/14
SKF38393 (*N* = 7 rats)	0/10	0/10	0/9	0/7
Quinpirole (*N* = 8 rats)	1/10	0/11	0/9	2/8

*Note*: Data presented as the number of cells (*n*) showing firing suppression over the total number of cells. *N* values representing the number of rats each dataset was recorded in are shown in the first column.

For MSNs which continued to fire action potentials in the presence of high dopamine concentrations (persistent firing MSNs), we investigated how their firing properties were altered. Both 30 µM and 100 µM dopamine significantly increased rheobase and decreased action potential firing frequency in the DLS (Table 7; Figure 5). These findings are consistent with a previous study (Akaike et al., 1987).

**TABLE 7:** Modulation of evoked action potential firing properties by dopamine (30 µM and 100 µM), SKF38393 (10 µM) and quinpirole (10 µM) in the dorsolateral striatum (DLS) and tail striatum (TS) divisions.

		Medial TS	Intermediate TS	Lateral TS	DLS	Two‐way ANOVA
Dopamine (30 µM) (*N* = 6 rats)	Rheobase (pA)	171 ± 99 *n* = 7	283 ± 174 *n* = 6	175 ± 75 *n* = 6	350 ± 155 *n* = 6	*P =* 0.00481 *F*(1, 21) = 9.95 ^**^
	Within‐region *P*‐value	0.2902	0.6207	0.5103	0.0161	
	Firing frequency (Hz)	13.9 ± 7.3	9.6 ± 3.6	13.3 ± 5.3	8.7 ± 3.0	*P =* 0.00492 *F*(1, 21) = 9.86 ^**^
	Within‐region *P*‐value	0.6846	0.0388	0.5024	0.0176	
	Delay to first action potential (s)	0.47 ± 0.34	0.19 ± 0.17	0.36 ± 0.31	0.19 ± 0.13	*P =* 0.893 *F*(1, 21) = 0.0184 NS
	Slope (Hz/pA)	0.13 ± 0.10	0.12 ± 0.07	0.04 ± 0.002	0.07 ± 0.02	*P =* 0.113 *F*(1, 21) = 2.73 NS
Dopamine (100 µM) (*N* = 11 rats)	Rheobase (pA)	180 ± 120 *n* = 9	245 ± 135 *n* = 13	189 ± 78 *n* = 13	300 ± 64 *n* = 6	*P <* 0.0001 *F*(1, 43) = 22.8 ^***^
	Within‐region *P*‐value	0.1486	0.0494	0.0141	0.0101	
	Firing frequency (Hz)	13.3 ± 5.0	10.8 ± 4.8	16.0 ± 5.6	10.2 ± 7.1	*P =* 0.00602 *F*(1, 43) = 8.37 ^**^
	Within‐region *P*‐value	0.3518	0.0197	0.7846	0.0240	
	Delay to first action potential (s)	0.32 ± 0.19	0.25 ± 0.16	0.21 ± 0.18	0.19 ± 0.12	*P =* 0.0168 *F*(1, 43) = 6.16 ^*^
	Within‐region *P*‐value	0.1354	0.3612	0.8694	0.0498	
	Slope (Hz/pA)	0.14 ± 0.10	0.08 ± 0.04	0.05 ± 0.001	0.03 ± 0.02	0.635 *F*(1, 43) = 0.228 NS
SKF38393 (*N* = 7 rats)	Rheobase (pA)	95 ± 47 *n* = 10	235 ± 167 *n* = 10	172 ± 67 *n* = 9	217 ± 102 *n* = 6	*P =* 0.0325 *F*(1, 31) = 5.01 ^*^
	Within‐region *P*‐value	>0.999	0.2431	0.2191	0.0488	
	Firing frequency (Hz)	10.4 ± 3.5	12.7 ± 4.4	13.5 ± 6.6	11.0 ± 3.2	*P =* 0.0356 *F*(1, 31) = 4.84 ^*^
	Within‐region *P*‐value	0.0456	0.4841	0.7625	0.2334	
	Delay to first action potential (s)	0.32 ± 0.21	0.27 ± 0.20	0.21 ± 0.15	0.25 ± 0.21	*P =* 0.208 *F*(1, 31) = 1.65 NS
	Slope (Hz/pA)	0.08 ± 0.04	0.07 ± 0.05	0.07 ± 0.05	0.06 ± 0.01	0.287 *F*(1, 31) = 1.17 NS
Quinpirole (*N* = 8 rats)	Rheobase (pA)	106 ± 84 *n* = 9	282 ± 152 *n* = 11	178 ± 100 *n* = 9	200 ± 102 *n* = 6	*P =* 0.00753 *F*(1, 30) = 8.19 ^*^
	Within‐region *P*‐value	0.9003	0.0101	0.3836	0.0360	
	Firing frequency (Hz)	12.8 ± 6.4	14.8 ± 4.8	12.6 ± 3.9	14.7 ± 3.4	*P =* 0.319 *F*(1, 30) = 1.03 NS
	Delay to first action potential (s)	0.20 ± 0.18	0.21 ± 0.16	0.37 ± 0.28	0.22 ± 0.16	*P =* 0.528 *F*(1, 30) = 0.407 NS
	Slope (Hz/pA)	0.05 ± 0.02	0.09 ± 0.05	0.06 ± 0.02	0.08 ± 0.04	0.514 *F*(1, 30) = 0.442 NS

*Note*: Firing frequency and delay to first action potential were measured at rheobase. *n* values representing the number of cells are shown in the first row for each condition and *N* values representing the number of rats each dataset was recorded in are shown in the first column. Data presented as means ± SD. Statistical significance of drug application was tested using repeated‐measures two‐way ANOVA and the *P‐* and *F*‐values reported. (NS *P >* 0.05,*P < 0.05, **P < 0.01, ***P < 0.0001). When significant, within‐region *P*‐values are also reported.

Within the TS, dopamine significantly decreased firing frequency in the intermediate division only, at both 30 µM and 100 µM. Both the intermediate and the lateral divisions showed increased rheobase by dopamine, but only at 100 µM. The medial TS showed no significant changes in firing properties in persistent firing MSNs. The delay to the first action potential and the slope of the current–frequency relationship were not altered by dopamine in any region.

Finally, persistent firing MSNs in the DLS showed extensive significant changes in action potential shape properties at both 30 µM and 100 µM dopamine, including peak amplitude, AHP amplitude, half‐width and time to peak (Appendix, Table A3). These findings are generally consistent with previous studies (Calabresi et al., 1987; Hu & Wang, 1988). In contrast, no significant changes in any action potential shape properties were identified in persistent firing TS‐MSNs.

#### 3.2.5 Modulation of action potential firing by D1‐ and D2‐agonism

We used the D1‐agonist SKF38393 (10 µM) and the D2‐agonist quinpirole (10 µM) to investigate the contribution of the D1 and D2 receptors to dopaminergic modulation in each TS division. Quinpirole induced firing suppression in a small number of MSNs in the medial TS and DLS only (Table 6), similar to 30 µM dopamine. Suppression of firing by quinpirole has been reported previously in the dorsal striatum (Perez et al., 2006). In contrast, SKF38393 did not cause firing suppression in any region.

In persistent firing neurons, quinpirole significantly affected rheobase (Table 7), but only in the DLS and the D2‐rich intermediate TS (Figure 5). Similarly, SKF38393 only had a significant effect in the DLS and the D1‐rich medial TS. Notably, SKF38393 significantly increased rheobase in the DLS (167 ± 25 pA to 217 ± 45 pA), whereas the medial TS showed significantly reduced firing frequency (14 ± 2 Hz to 10 ± 1 Hz). The effects of quinpirole (Hu & Wang, 1988; Perez et al., 2006) and SKF38393 (Akaike et al., 1987; Schiffmann et al., 1995) in the DLS were consistent with previous work. Both quinpirole and SKF had no significant effect on action potential shape properties (Appendix, Table A3).

#### 3.2.6 Division‐based electrophysiological classification of TS‐MSNs

We successfully classified MSNs as medial division (versus other) with MCC = 0.52 and accuracy = 76.45% using the following features: rheobase, firing frequency, EPSC frequency. This performance was statistically significantly above chance (*P* < 0.05), as per our shuffle test, which, using these features, produced MCC values ranging from −0.12 to 0.21 (95% confidence interval, CI) and accuracy ranging from 69.5% to 73% (95% CI).

MSNs were also successfully classified as lateral division (versus other) with MCC = 0.32 and accuracy = 74.67% using rheobase, firing frequency and membrane resistance. This performance was significantly above chance; our shuffle test using these features produced MCC values ranging from −0.21 to 0.19 (95% CI) and accuracy ranging from 55% to 65% (95% CI).

Kruskal–Wallis tests did not show statistically significant variation in EPSC frequency or membrane resistance across divisions. Thus, classification appears to be a useful method for discriminating divisions by capturing the subtle interactions of multiple physiological features.

MSNs were not successfully classified as intermediate division (versus other) at rates above chance. This failure of classification could reflect greater variability among individual MSNs within the intermediate division relative to the medial or lateral divisions.

## 4 DISCUSSION

Here, we carried out the first in‐depth electrophysiological characterisation of MSNs in the TS divisions. TS‐MSNs were electrophysiologically distinct from DLS‐MSNs, reflecting the functional differences between these domains (Alegre‐Cortés et al., 2021; Riley et al., 2024; Todd et al., 2022). The TS divisions also exhibited differences in action potential firing, supporting that they, too, are functionally distinct. Finally, differences in dopaminergic modulation were identified between the DLS and TS, and across the TS divisions, which could have implications for dopaminergic disease.

### 4.1 TS‐MSN electrophysiology and comparison to DLS‐MSNs

We found TS‐MSNs to have membrane properties typical of MSNs elsewhere in the striatum (Hammond, 2015; Tepper et al., 1998; Ting et al., 2024; Venance & Glowinski, 2003), except for their RMP, which was more depolarised than expected (−73 ± 1 mV). DLS MSNs had more typical RMPs (−79 ± 1 mV), supporting that TS‐MSNs’ depolarisation is physiological rather than an indicator of poor neuron health (Hammond, 2015). MSNs usually maintain deeply hyperpolarised RMPs through powerful rectifying currents (Mermelstein et al., 1998). While TS‐ and DLS‐MSNs exhibited similar rectifying currents upon hyperpolarisation from −80 mV, they may have subtle differences in rectification at rest, which cause TS‐MSNs to be more depolarised (Mermelstein et al., 1998).

TS‐MSNs were more excitable than DLS‐MSNs. This finding is consistent with a recent report comparing a small sample of TS‐MSNs to caudal dorsal striatum MSNs in mice (Ogata et al., 2022). This difference in excitability possibly reflects heterogeneity in the density and subtypes of voltage‐gated sodium and potassium channels expressed by MSNs, which are involved in action potential generation and largely determine firing properties (Arnsdorf & Makielski, 2001). This heterogeneity is supported by the differences in action potential shape identified between the TS and DLS. TS‐MSNs’ higher excitability may also relate to their lower capacitance (Taylor, 2012), which allows the membrane to charge faster and require less charge to depolarise. Finally, differences in MSN neuromodulation between the TS and DLS, including by dopamine (Valjent & Gangarossa, 2021) and local interneurons (Miyamoto et al., 2019), could also contribute to uneven excitability between the TS and DLS (Gertler et al., 2008; Oldenburg & Ding, 2011).

Capacitance reflects membrane surface area but is weighted toward the site of voltage clamping (i.e. the soma and proximal projections; Taylor, 2012). Because TS‐MSNs had larger somas than DLS‐MSNs, we propose that their lower capacitance reflects reduced dendritic arborisation (Taylor, 2012). Reduced arborisation can be indicative of lower glutamatergic input, which powerfully governs dendritic growth (Cline & Haas, 2008); however, TS‐MSNs received more glutamatergic EPSCs than DLS‐MSNs, suggesting the opposite. The tail divisions are indicated to receive distinct inputs (Miyamoto et al., 2018; Ogata et al., 2022) and may have dendrites confined within divisions, restricting arborisation due to the divisions’ narrow shape (Yelnik et al., 1994). The high frequency and amplitude of TS‐MSN EPSCs relative to DLS‐MSN EPSCs indicates a higher density of synaptic input, or greater release probability in individual synapses (Flight, 2008; Golowasch et al., 2009). TS‐projecting dopamine neurons are also proposed to co‐release glutamate (Menegas et al., 2015), which could contribute to TS‐MSN EPSCs and regulate TS‐MSNs alongside dopamine. Combined, these differences show that TS‐MSNs are distinct from DLS‐MSNs and suggest that TS‐MSNs have high sensitivity to synaptic inputs, which could be beneficial for sensory integration.

### 4.2 Comparison of the TS divisions

We showed differences in action potential firing between the TS divisions. Functional heterogeneity between the divisions is further supported by our classification analysis, which shows that the medial and lateral divisions have unique electrophysiological signatures. Features driving classification were primarily related to action potential firing, highlighting that this is where TS‐MSNs diverge. Notably, intermediate division TS‐MSNs lacked a robust electrophysiological signature, suggesting greater variability among individual MSNs, potentially due to the presence of MSN subtypes beyond D1‐ and D2‐MSNs, as shown elsewhere in the striatum (Stanley et al., 2020).

Lateral division TS‐MSNs were more excitable (lower rheobase, higher firing frequency) than TS‐MSNs in the medial and intermediate divisions and fired slightly larger action potentials than TS‐MSNs in the medial division. Given that membrane properties were uniform across the TS, we propose that this regional variation arises from differences in voltage‐gated sodium and potassium currents between divisions (Arnsdorf & Makielski, 2001). Reduced local inhibition could also contribute, as inhibitory interneurons are abundant in the TS but least dense in the lateral division (Miyamoto et al., 2019).

TS‐MSNs in the intermediate division and medial division also exhibited a small difference in rheobase but otherwise had consistent firing and membrane properties. This small difference could arise from subtle differences in voltage‐gated sodium currents during action potential generation, such as in voltage‐gating thresholds (Calabresi et al., 1987; Tkatch et al., 2000). Because dopamine powerfully modulates MSN excitability, distinct dopamine receptor expression (Ogata et al., 2022) and transmission properties (Riley et al., 2024) between the medial and intermediate divisions could also contribute.

Interestingly, TS‐MSNs in the lateral division also uniquely showed modulation of EPSCs by quinpirole, supporting that corticothalamic terminals in this division, but not in the medial or intermediate divisions, express D2 receptors. These D2‐expressing corticothalamic terminals could arise from input sources specific to the lateral division, such as the agranular insula cortex, which has been shown to have inputs to the lateral division that are not shared with the other TS divisions (Miyamoto et al., 2018).

#### 4.2.1 Electrophysiological similarity of the medial and intermediate TS divisions

We found TS‐MSNs in the D1‐rich medial and D2‐rich intermediate divisions to have similar resting membrane properties, consistent with Ogata et al. (2022). In contrast, rostral striatum D1‐ and D2‐MSNs exhibit extensive differences in resting membrane properties, including RMP, membrane resistance and membrane time constant (Chuhma & Rayport, 2024; Francis et al., 2015; Gertler et al., 2008). The small proportion (approximately 10−20%; Ogata et al., 2022) of D2‐ and D1‐MSNs in the medial and intermediate divisions, respectively, may have masked differences arising from the uneven D1/D2 distribution. However, with the large number of neurons recorded from each division and the substantial differences between D1‐ and D2‐MSNs reported previously, any masking effect should be small. These findings indicate that D1‐ and D2‐MSNs in the TS are more similar to each other than in the rostral striatum, despite their segregated organisation; in this regard, the TS differs from the rostral striatum.

### 4.3 Distinct dopaminergic modulation of action potential firing between the DLS and TS

We found that dopamine broadly inhibited action potential firing across the DLS and all TS divisions, but each region showed a distinct pattern of modulation. TS‐MSNs were less sensitive to modulation by dopamine (30 µM and 100 µM) than DLS‐MSNs. Differences in dopamine receptor expression (Ogata et al., 2022) and transmission properties (Riley et al., 2024) between the medial and intermediate TS and DLS could contribute to this uneven sensitivity. However, the lateral TS, which shows dopamine receptor expression (approximately 1:1; Ogata et al., 2022) and transmission properties (Riley et al., 2024) comparable to the DLS, also showed comparatively low dopamine sensitivity. Furthermore, while DLS‐MSNs showed modulation by both SKF38393 and quinpirole, in the TS, only the D2‐rich intermediate division responded to quinpirole, only the D1‐rich medial division responded to SKF38393, and the lateral division responded to neither. We propose that these differences in sensitivity arise from TS‐MSNs expressing fewer dopamine receptors than DLS‐MSNs. This is supported by Ogata et al. (2022), who reported similar D1 expression (using quantified immunofluorescence) across the medial TS and DLS, and similar D2 expression across the intermediate TS and DLS, despite these TS divisions containing substantially higher proportions of D1‐ and D2‐MSNs, respectively, than the DLS, and a comparable density of MSNs. It is also possible that dopamine receptors in the TS have a weaker functional effect (weaker coupling to downstream effectors) than those in the DLS, but this remains to be tested.

#### 4.3.1 Heterogeneity in dopaminergic modulation of firing across the TS divisions

The prevalence of dopamine‐induced firing suppression was uneven across the TS divisions, most common in the medial division, rare in the lateral division, and absent in the intermediate division. Previous work has indicated that dopamine‐induced suppression of firing is D2‐dependent (Brown & Arbuthnott, 1983; Calabresi et al., 1987). Consistent with this, we found that dopamine and quinpirole, but not SKF38393, induced firing suppression in a subset of MSNs. Thus, the high prevalence of dopamine‐induced firing suppression in the D2‐poor medial division and its absence in the D2‐rich intermediate division were unexpected. This finding could be explained by heterogeneity in the functional effects of D2 activation across regions, particularly in the modulation of A‐type potassium currents, from which D2‐mediated firing suppression is thought to arise (Perez et al., 2006). Regional differences in off‐target interactions, such as via adrenergic receptors (Hara et al., 2010) or dopaminergic activation of interneurons (Bracci et al., 2002), could also contribute.

The lateral division showed minimal dopaminergic firing suppression and modulation of persistent firing MSNs compared to the medial and intermediate divisions. This may relate to the medial and intermediate divisions having lower dopamine transmission than the lateral division (Riley et al., 2024), potentially driving increased dopamine sensitivity. Supporting this, in the intermediate division, where dopaminergic transmission is particularly low (Riley et al., 2024), persistent firing TS‐MSNs showed the greatest dopaminergic modulation of firing. Differences in dopamine receptor expression between the TS divisions (Ogata et al., 2022) likely also contributes to their heterogeneous dopamine sensitivity.

The intermediate division was the only region within the TS in which persistent firing MSNs showed altered firing by quinpirole, reflecting the enrichment of D2 in this region (Ogata et al., 2022). As seen in the DLS, quinpirole increased rheobase in the intermediate TS, likely through similar mechanisms (modulation of BK and delayed rectifier channels; Ji & Martin, 2014).

Similarly, the D1‐rich medial division (Ogata et al., 2022) was the only region within the TS in which SKF38393 altered firing. However, the effect of SKF38393 in the medial division (reduced firing frequency) differed from that in the DLS (increased rheobase), suggesting distinct functional effects of D1 activation. SKF38393‐induced firing inhibition in the DLS is attributed to the enhancement of rectifying currents (Pacheco‐Cano et al., 1996) and downregulation of voltage‐gated sodium currents (Calabresi et al., 1987; Ji & Martin, 2014; Schiffmann et al., 1995; Surmeier et al., 1992). In contrast, the medial TS may show greater D1‐mediated modulation of A‐type potassium channels, which powerfully shape firing frequency (Tkatch et al., 2000). Differences in intrinsic firing properties between the DLS and medial TS could also play a role.

### 4.4 Functional significance

TS‐MSNs’ high sensitivity to synaptic input, similar to that in the ventral striatum (Chuhma & Rayport, 2024), facilitates the capturing and processing of subtle or transient signals, which would be beneficial for the sensory integration and processing carried out by this domain (Nadasdy, 2009). Conversely, DLS‐MSNs’ low sensitivity confers high‐fidelity signalling, beneficial for robust motor commands (Mahon et al., 2000).

We identified electrophysiological differences between the TS divisions, supporting that they are functionally heterogeneous. Prior anatomical findings show differences in the cortical input to each TS division (Miyamoto et al., 2018), which could impose specialised functional roles on each division. The distinct electrophysiological properties of each division may facilitate these specialised functions. Further characterisation of the specific network connections of each division would provide important insight into their specific functions (Lee et al., 2023).

Striatal hyperdopaminergia has well‐established connections with sensory symptoms like hallucinations (Brisch et al., 2014; Weil & Reeves, 2020), including in schizophrenia (Laruelle et al., 1999) and levodopa‐induced Parkinson's disease psychosis (Moskovitz et al., 1978). Recent evidence implicates the TS as a key locus in dopamine's role in hallucinations (Lakshminarasimhan et al., 2025) and shows that increases in TS dopamine can induce hallucination‐like events in mice (Schmack et al., 2021). Here, we showed that bath‐applying dopamine in vitro induces changes in the firing properties of TS‐MSNs, including at 30 µM, which is comparable to physiological concentrations during levodopa‐induced dopamine surges (Mosharov et al., 2015) associated with hallucinations (Moskovitz et al., 1978). Notably, dopaminergic modulation of firing was uneven between the medial and intermediate divisions, which have primarily direct and indirect pathway output, respectively and may function cooperatively to provide balanced outputs to downstream regions. Loss of balanced direct and indirect pathway output from the DLS in dopaminergic disease is well established to drive motor symptoms (Lanciego et al., 2012). Thus, uneven modulation of the medial and intermediate TS divisions in dopaminergic disease could contribute to sensory symptoms by disrupting the balance of their pathway outputs. Further investigation into how the medial and intermediate divisions modulate downstream regions, cooperatively or independently, and how this network is altered by elevated dopamine, could provide important insights into how the TS contributes to sensory symptoms in dopaminergic disease and how the TS could be targeted therapeutically.

## AUTHOR CONTRIBUTIONS

Conception and design of the work: Bronwyn Riley, Peter S. Freestone, Johanna M. Montgomery, Srdjan M. Vlajkovic. Acquisition, analysis or interpretation of data for the work: Bronwyn Riley, Peter S. Freestone, Luke E. Hallum. Drafting the work or revising it critically for important intellectual content: Bronwyn Riley, Peter S. Freestone, Luke E. Hallum, Srdjan M. Vlajkovic, Johanna M. Montgomery. All authors have approved the final version of the manuscript and agree to be accountable for all aspects of the work in ensuring that questions related to the accuracy or integrity of any part of the work are appropriately investigated and resolved. All persons designated as authors qualify for authorship, and all those who qualify for authorship are listed.

## CONFLICT OF INTEREST

None declared.

## GENERATIVE AI STATEMENT

No generative AI has been used in the preparation of this manuscript.

## Data Availability

Data are available on request from the authors. The custom MATLAB code used to implement Fisher's linear discriminant analysis, classifier training and performance evaluation is also available upon request.

## 1

**TABLE A1:** Paired regional comparison of electrophysiological properties between tail striatum (TS) divisions.

	TS division pairs	Multiple‐comparisons *P*‐value
Short axis length	Medial intermediate	0.0664
	Meidal lateral	0.506
	Intermediate lateral	0.275
Short axis: long axis	Medial intermediate	0.968
	Meidal lateral	0.432
	Intermediate lateral	0.359
RMP	Medial intermediate	0.327
	Meidal lateral	0.962
	Intermediate lateral	0.394
Tau	Medial intermediate	0.237
	Meidal lateral	0.649
	Intermediate lateral	0.507
EPSC amplitude	Medial intermediate	0.644
	Meidal lateral	0.752
	Intermediate lateral	0.885
EPSC frequency	Medial intermediate	0.771
	Meidal lateral	0.754
	Intermediate lateral	0.521
Rheobase	Medial intermediate	0.00681
	Meidal lateral	>0.9999
	Intermediate lateral	0.000700
Firing frequency	Medial intermediate	>0.9999
	Meidal lateral	0.0196
	Intermediate lateral	0.00100
Peak amplitude	Medial intermediate	0.477
	Meidal lateral	0.0258
	Intermediate lateral	>0.9999

Only properties in which statistically significant (*P <* 0.05) regional variation was identified by Kruskal–Wallis tests are included. Multiple‐comparisons *P*‐values are shown across all TS division pairs.

**TABLE A2:** Modulation of spontaneous excitatory postsynaptic currents (EPSC) by dopamine (30 µM, 100 µM) and SKF38393 (10 µM) in the dorsolateral striatum (DLS) and tail striatum (TS) divisions.

	Medial TS	Intermediate TS	Lateral TS	DLS	Two‐way ANOVA
Dopamine (30 µM) EPSC amplitude (pA) (*N* = 6 rats)	−14.2 ± 9.2 *n* = 9	−21.6 ± 6.9 *n* = 6	−14.0 ± 5.6 *n* = 6	−17.5 ± 5.1 *n* = 7	*P =* 0.473 *F*(1, 45) = 1.48 NS
Dopamine (30 µM) EPSC frequency (Hz)	4.6 ± 1.7	3.1 ± 1.0	4.6 ± 1.1	2.6 ± 0.6	*P =* 0.259 *F*(1, 45) = 1.35 NS
Dopamine (100 µM) EPSC amplitude (pA) (*N* = 11 rats)	−19.4 ± 4.7 *n* = 12	−14.9 ± 5.6 *n* = 13	−20.2 ± 7.1 *n* = 15	−14.2 ± 6.0 *n* = 14	*P =* 0.473 *F*(1, 61) = 0.528 NS
Dopamine (100 µM) EPSC frequency (Hz)	2.9 ± 1.2	2.6 ± 1.3	2.2 ± 0.9	1.9 ± 0.6	*P =* 0.249 *F*(1, 61) = 1.36 NS
SKF38393 EPSC amplitude (pA) (*N* = 7 rats)	−14.2 ± 3.5 *n* = 10	−15.5 ± 5.1 *n* = 10	−15.1 ± 6.9 *n* = 9	−17.5 ± 5.5 *n* = 7	*P =* 0.193 *F*(1, 31) = 1.83 NS
SKF38393 EPSC frequency (Hz)	4.1 ± 2.6	3.4 ± 2.0	3.1 ± 1.6	2.4 ± 1.7	*P =* 0.747 *F*(1, 31) = 0.106 NS

*n* values representing the number of cells are shown in the first row for each condition. *N* values representing the number of rats each dataset was recorded in are shown in the first column. Data presented as mean ± SD. Statistical significance of the drug effect was tested using repeated‐measures two‐way ANOVA and the *P‐* and *F*‐values reported. (NS *P >* 0.05).

**TABLE A3:** Modulation of evoked action potential shape properties by dopamine (30 µM, 100 µM), SKF38393 (10 µM) and quinpirole (10 µM) in the dorsolateral striatum (DLS) and tail striatum (TS) divisions.

Drug		Medial TS	Intermediate TS	Lateral TS	DLS	Two‐way ANOVA
Dopamine (30 µM) (*N* = 6 rats)	Peak amplitude (mV)	67.9 ± 13.0 *n* = 7	63.3 ± 6.6 *n* = 6	71.4 ± 11.2 *n* = 6	57.9 ± 13.9 *n* = 6	*P <* 0.0001 *F*(1, 21) = 21.8 ^***^
	Within‐region *P*‐value	0.278	0.205	0.203	<0.0001	
	AHP amplitude (mV)	−8.8 ± 5.9	−7.2 ± 3.9	−9.3 ± 2.4	−4.6 ± 2.6	*P =* 0.0170 *F*(1, 21) = 6.72 ^*^
	Within‐region *P*‐value	0.672	0.720	0.734	<0.0001	
	Time to peak (ms)	1.56 ± 0.61	1.61 ± 0.29	1.83 ± 0.53	1.9 ± 0.26	*P =* 0.0603 *F*(1, 21) = 5.93 NS
	Half‐width (ms)	3.02 ± 1.34	3.18 ± 0.44	3.86 ± 0.58	2.69 ± 0.78	*P =* 0.199 *F*(1, 21) = 1.76 NS
Dopamine (100 µM) (*N* = 11 rats)	Peak amplitude (mV)	70.3 ± 15.7 *n* = 9	66.8 ± 10.3 *n* = 13	63.3 ± 14.4 *n* = 13	62.7 ± 17.1 *n* = 6	*P =* 0.019 *F*(1, 43) = 5.89 ^*^
	Within region *P*‐value	0.668	0.775	0.0729	0.100	
	AHP amplitude (mV)	−9.0 ± 3.7	−10.2 ± 3.7	−10.4 ± 3.6	−5.0 ± 3.0	*P =* 0.000310 *F*(1, 43) = 15.4 ^**^
	Within‐region *P*‐value	0.0541	0.166	0.748	0.000401	
	Time to peak (ms)	1.64 ± 0.27	1.51 ± 0.45	1.73 ± 0.60	2.18 ± 0.66	*P =* 0.792 *F*(1, 43) = 0.0704 NS
	Half‐width (ms)	4.10 ± 1.11	4.14 ± 1.18	4.27 ± 1.00	4.53 ± 0.33	*P =* 0.0449 *F*(1, 43) = 4.33 ^*^
	Within‐region *P*‐value	0.841	0.904	0.510	0.0005	
SKF38393 (*N* = 7 rats)	Peak amplitude (mV)	69.8 ± 9.2 *n* = 10	67.8 ± 11.6 *n* = 10	72.0 ± 13.5 *n* = 9	70.3 ± 12.5 *n* = 6	*P =* 0.0575 *F*(1, 31) = 3.89 NS
	AHP amplitude (mV)	−6.2 ± 3.0	−7.0 ± 4.0	−8.8 ± 3.8	−6.4 ± 2.8	*P =* 0.0206 *F*(1, 31) = 1.67 NS
	Time to peak (ms)	1.54 ± 0.24	1.59 ± 0.41	1.52 ± 0.39	1.65 ± 0.27	*P =* 0.367 *F*(1, 31) = 0.839 NS
	Half‐width (ms)	2.89 ± 0.85	3.51 ± 1.08	3.07 ± 1.05	2.94 ± 0.56	*P =* 0.349 *F*(1, 31) = 0.903 NS
Quinpirole (*N* = 8 rats)	Peak amplitude (mV)	67.5 ± 10.3 *n* = 9	68.6 ± 13.3 *n* = 11	67.5 ± 8.6 *n* = 9	70.0 ± 12.5 *n* = 6	*P =* 0.341 *F*(1, 30) = 0.936 NS
	AHP amplitude (mV)	−10.3 ± 6.1	−5.9 ± 3.6	−10.0 ± 5.8	−8.6 ± 4.2	*P =* 0.360 *F*(1, 30) = 0.863 NS
	Time to peak (ms)	1.86 ± 0.61	1.71 ± 0.25	1.28 ± 0.35	1.84 ± 0.51	*P =* 0.749 *F*(1, 30) = 0.104 NS
	Half‐width (ms)	3.04 ± 1.05	3.10 ± 0.98	2.82 ± 1.01	2.98 ± 1.09	*P =* 0.445 *F*(1, 30) = 0.600 NS

Properties were measured from the first action potential fired at rheobase. *n* values representing the number of cells and *N* values representing the number of rats each dataset was recorded in are shown in the first row for each condition. Data presented as the means ± SD. Statistical significance of drug application was tested using repeated‐measures two‐way ANOVA and the *P‐* and *F*‐values reported. (NS *P >* 0.05,**P <* 0.05, ***P <* 0.01, ****P <* 0.0001). When significant, within‐region multiple comparisons *P*‐values are also reported.
